# A mother’s gift: Provisioning of plastid-derived structures into eggs promotes invertebrate development and dispersal

**DOI:** 10.1371/journal.pbio.3003753

**Published:** 2026-04-24

**Authors:** Jillian P. Lewis, Spencer V. Nyholm

**Affiliations:** Department of Molecular and Cell Biology, University of Connecticut, Storrs, Connecticut, United States of America

## Abstract

Eggs released in the environment are at risk from many threats. This Primer discusses how plastid-like carotenoid crystals benefit larval survival and trans-oceanic dispersal in sea urchin eggs.

## Teaser

Eggs released in the environment are at risk from many threats. A recent study in *PLOS Biology* reveals that plastid-derived carotenoid crystals in sea urchin eggs benefit larval survival and *trans-*oceanic dispersal.

Development can be challenging, especially for animals that release their eggs or deposit them in the environment. External eggs must be protected from predation, fouling, and pathogens. Some animals deposit bacteria or bacterial products directly into their eggs to provide antimicrobials or other defenses that protect the developing embryos from pathogens or fouling [1,2]. Some animals also use symbiotic microbes to provide metabolic or nutritional components to assist in the development of their offspring. For example, yellow-spotted salamander eggs have symbiotic algae that deliver fixed carbon and oxygen and promote embryonic development [3]. Insects including fruit flies and honey bees host symbiotic bacteria that provide amino acids to their larvae, aiding in larval growth and development [4,5]. These adaptations can help ensure reproductive success and survival of the next generation.

Marine organisms that engage in broadcast spawning or dispersal into the water column are often at the mercy of oceanic currents. Eggs and larvae may travel long distances (tens to thousands of kilometers) before development is complete. Although phytoplankton, dissolved organic matter, and bacteria can help sustain these larvae on their journey, there is often high mortality [6]. However, the planktotrophic (plankton-consuming) larvae of some organisms have evolved additional mechanisms to overcome this challenge and ensure successful dispersal and survival.

**Fig 1:**
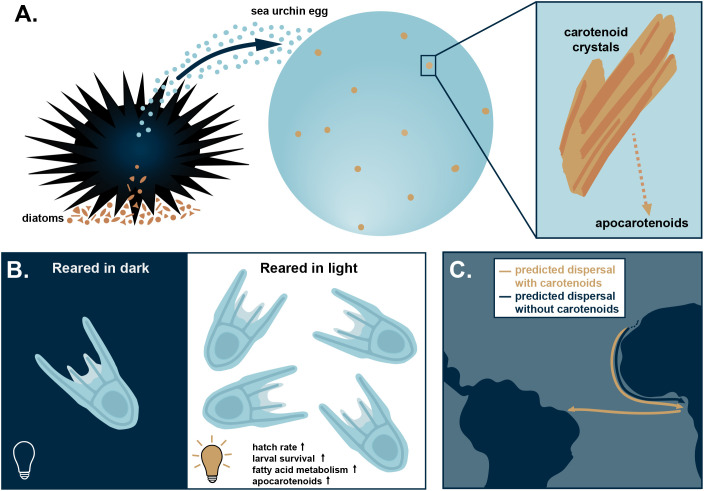
Transmission and benefits of plastid-derived structures in sea urchin eggs. **(A)** Proposed mechanism of transmission of plastid-derived structures. From left to right: Adult female sea urchin ingests diatoms and releases eggs that contain carotenoid-like crystals. These carotenoid crystals may be enzymatically cleaved into apocarotenoids (i.e., phytohormones). **(B)** Eggs of *Arbacia lixula* reared in light (receiving benefits from carotenoids) had higher hatching and survival rates, increased fatty acid metabolism, and an enrichment of apocarotenoids compared to those reared in the dark. **(C)** Larvae receiving benefits from carotenoids were modeled to disperse along the African shelf in fewer generations and predicted to survive trans-oceanic dispersal.

In this issue of *PLOS Biology*, Carrier and colleagues present evidence that components of plastids (membrane-bound organelles such as chloroplasts) are present in sea urchin eggs [7]. They further provide evidence suggesting that these plastid components may promote the development and survival of larvae during marine dispersal (Fig 1). Animals that ingest photosynthetic organisms can incorporate plastids into their tissues in a phenomenon referred to as kleptoplasty (e.g., sacoglossan sea slugs) [8]. Animals that consume plastids are also exposed to non-photosynthetic plastid derivatives called chromoplasts that contain carotenoids. Many adult sea urchins graze on microscopic algae such as diatoms and are thereby exposed to plastids and chromoplasts through their diet.

A meta-analysis of 16S rRNA gene sequences from eggs of a dozen sea urchin species revealed that in addition to unique bacterial communities, eggs from planktotrophic sea urchin species contain plastid DNA originating from diatoms and other photosynthetic eukaryotes. Interestingly, no plastid sequences were found in the only lecithotrophic (nonfeeding larvae) sea urchin species analyzed; eggs from this group receive a larger yolk provision to sustain them. Focusing on a planktotrophic species, the black sea urchin *Arbacia lixula*, the authors found that the eggs contained 16S rRNA genes derived from plastids, whereas the 18S rRNA gene from other eukaryotic nuclei was absent. Fluorescence and transmission electron microscopy of unfertilized *A. lixula* eggs revealed that most eggs contained particles of crystallized carotenoid pigments. These particles are not full chromoplasts since there is no evidence of a membrane compartmentalizing the crystals from the egg cytoplasm. The authors propose that these carotenoid crystals are derived from chromoplast components that result from ingested diatoms and are transferred to eggs by mother sea urchins during oogenesis.

Carotenoid crystals in the eggs may be a strategy to benefit larval survival while maintaining high fecundity in a challenging environment. Carotenoid crystals in chromoplasts are involved in light-dependent reactions that can benefit host fitness. For example, when experimentally fed carotenoids, adult sea urchin survival and offspring size is improved [9]. When *A. lixula* eggs were maintained in light and dark conditions without feeding, the authors found that eggs kept in the light were more likely to develop into larvae and had better survival after hatching. Larvae that developed in the dark displayed a morphological phenotype consistent with nutritional restriction. The metabolic profile of larvae also differed between the two conditions, with an overall shift in fatty acid metabolism and enriched apocarotenoids (i.e., phytohormones) under light conditions. The survival data from light and dark experiments was then used to model predictions about how far these larvae might disperse when benefitting from a metabolic boost provided by the carotenoid crystals. Based on ocean currents, the model predicted that larvae that benefit from chromoplast-derived structures live longer and disperse farther. These larvae were predicted to survive dispersal across the Atlantic Ocean to South America, a journey that *A. lixula* larvae make yearly from the species’ southern range in Africa.

The results from this study suggest that chromoplast components in eggs benefit larvae. However, much of the evidence of larval survival and dispersal is based on a correlation: that light is synonymous with benefits from carotenoid metabolism. The mechanistic link between carotenoid metabolism and development also remains to be characterized. While carotenoids in chromoplasts can be involved in light-dependent reactions, carotenoids can also be converted into apocarotenoids as a part of animal and microbial metabolism [10]. Apocarotenoids were enriched in light-reared larvae, so further research may link the functional capabilities of sea urchins or the metagenomes of egg-associated microbes to carotenoid metabolism.

On a broader scale, evidence of plastid-derived structures in sea urchin eggs suggests that *A. lixula* may receive these plastid remnants from their mother as an investment in egg and larval survival. This is a novel discovery—no other animal has been found to provision plastid-derived structures to the germ line to promote larval development and survival. However, there are still many unanswered questions about the origin of these plastid-derived structures. The authors proposed a potential mechanism: adult sea urchins ingest photosynthetic diatoms and other eukaryotes, whose chloroplasts differentiate to non-symbiotic chromoplasts inside the adult sea urchin’s body cavity. The resulting chromoplast-derived carotenoid crystals are then incorporated into the sea urchin eggs where light-dependent reactions and metabolites give the larvae an advantage. The results of this study reveal a previously undescribed mode of maternal provisioning and help establish A. *lixula* as an emerging model to explore understudied mechanisms of marine larval dispersal and survival.
